# An Unusual Case Report of Male Genital Tuberculosis

**DOI:** 10.7759/cureus.64950

**Published:** 2024-07-19

**Authors:** Tamizharasan Masilamani, Nalini Jayanthi, Anitha Elaiyalwar, Karniha B, Shivasekar Ganapati

**Affiliations:** 1 Respiratory Medicine, SRM Medical College Hospital and Research Centre, Chennai, IND; 2 Pathologist, SRM Institute of Science and Technology, Chennai, IND

**Keywords:** langhans giant cell, acid fast bacilli (afb), testicular tuberculosis, extra pulmonary tuberculosis, genital tuberculosis

## Abstract

The burden of extrapulmonary tuberculosis (EPTB) is significant, constituting up to 20% of all TB cases in HIV-negative patients and 50% of new cases in HIV-positive individuals. However, diagnosing EPTB remains challenging due to its pauci-bacillary nature and the necessity for invasive sampling methods in many forms of the disease. Urogenital tuberculosis represents approximately 4% of the annual cases of extra-pulmonary tuberculosis in India, with isolated testicular tuberculosis being a particularly rare manifestation. In this report, we present three cases of testicular tuberculosis, diagnosed through tissue biopsy and Acid Fast Bacilli (AFB) smears.

## Introduction

Tuberculosis (TB), caused by *Mycobacterium tuberculosis*, can affect any tissue or organ in the body. Extrapulmonary TB (EPTB) accounted for 16% of the 7.5 million reported TB cases worldwide and 19% of cases in Southeast Asia [1,2]. Urogenital TB encompasses TB infections of the female and male reproductive systems as well as the urinary system. This condition often progresses slowly and may manifest with different symptoms depending on the location and progression of the infection [3]. Urogenital TB is categorized into three main types: (1) urinary TB, which affects the kidneys, ureters, and bladder; (2) female genital TB, which targets the uterus, fallopian tubes, and ovaries; and (3) male genital TB, which involves the epididymis and/or testes [4]. The purpose of this case report was to describe the clinical features of unilateral testicular TB and to underline that EPTB should be considered among other differential diagnoses.

## Case presentation

Case 1

**Figure 1:**
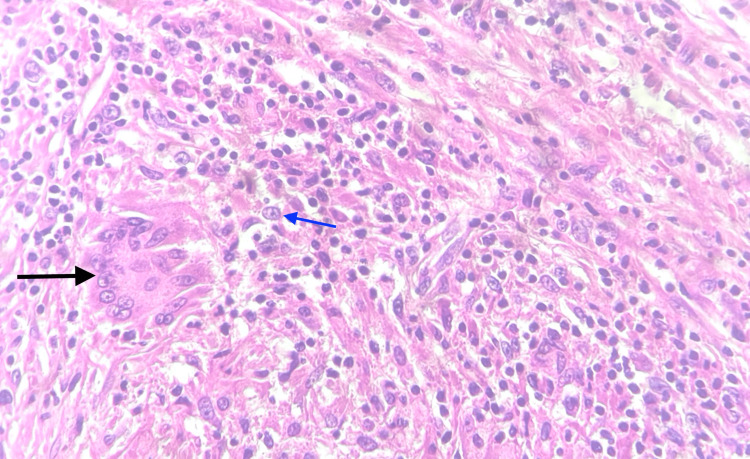
H&E 40× showing orchitis with granuloma and Langhans giant cells (black arrow) with lymphocytes and epithelioid cells (blue arrow) background

**Figure 2:**
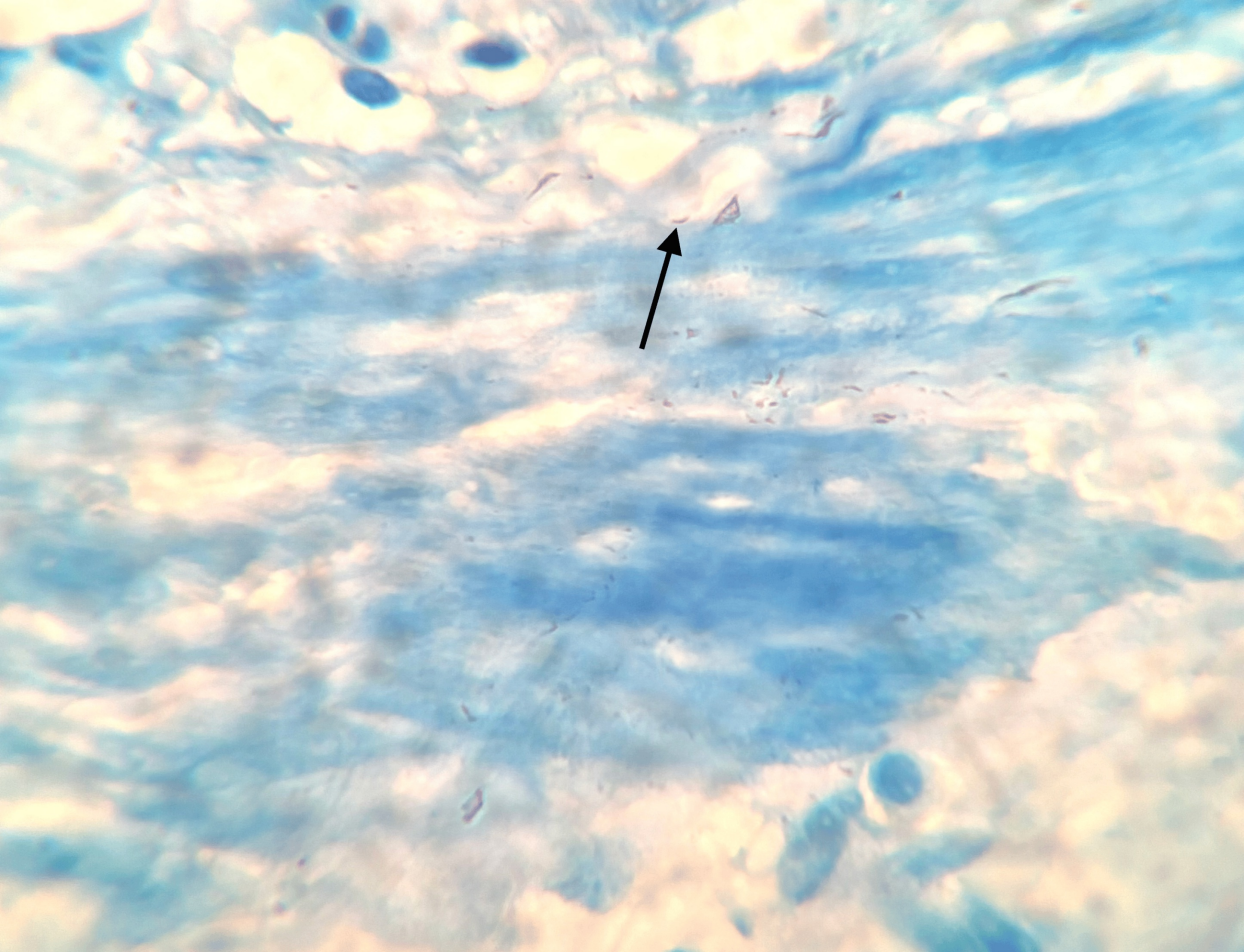
Oil immersion with 100×-modified Ziehl-Neelsen stain showing Acid Fast Bacilli (black arrow)

A 76-year-old man presented with a six-month history of swelling and pain on the left side of his scrotum and a low-grade fever for two weeks. On examination, firm to hard nodules are present in the left testis. CT kidney, ureter, and bladder (KUB) contrast showed a well-defined peripheral enhanced lesion measuring 1.8 × 1.3 × 1.3 cm in the left testis with central non-enhancing areas suggestive of necrosis. Histopathology (Figure 1) showed granuloma with epithelioid histiocytes and Langhans giant cells with necrosis. A left orchidectomy was done, and the biopsy specimen (Figure 2) was positive for Acid Fast Bacilli (AFB).

Case 2

**Figure 3:**
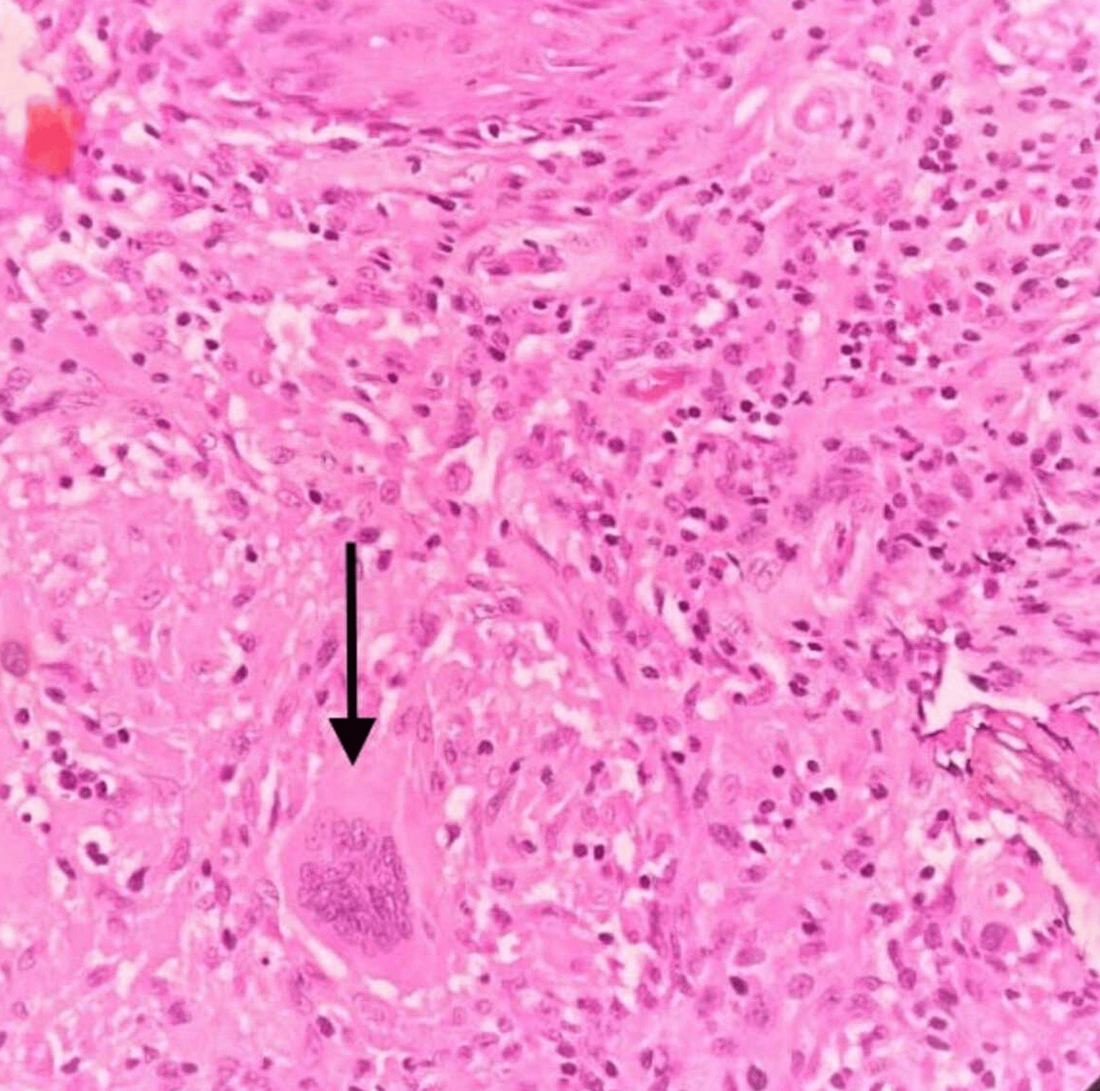
H&E 40× showing histiocytic aggregates forming granulomas surrounded by lymphocytes and Langhans giant cell (black arrow)

**Figure 4:**
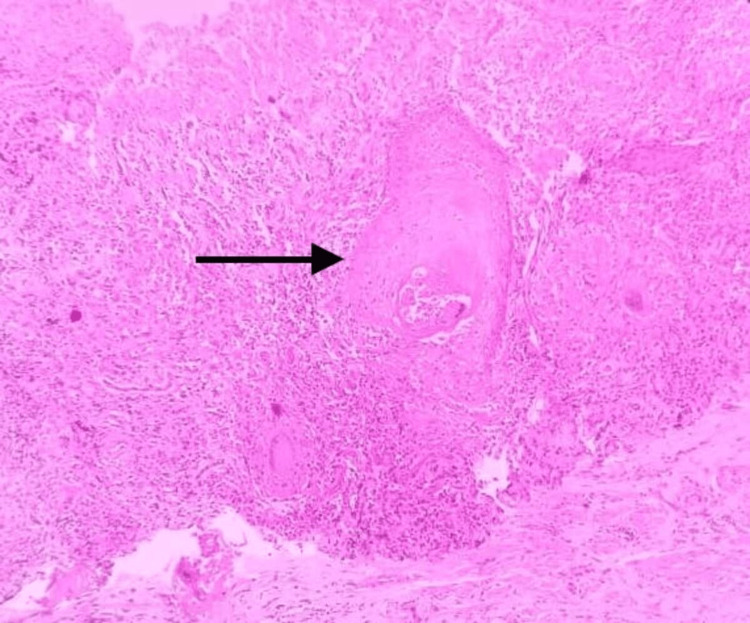
H&E 40× showing caseous necrosis (black arrow)

A 35-year-old man reported experiencing swelling on the left side of his scrotum for the last seven months and a low-grade fever for seven months. The swelling gradually increased in size, and the patient developed a non-radiating, diffuse type of pain with pustules over the left scrotal wall, which spontaneously burst with scanty discharge followed by another pustule around the same area. CT KUB contrast showed a cyst of 1 cm in the left testis with a sinus tract of 1.5 cm extending into the left scrotal wall. Excision of the sinus tract and cyst was done. Histopathological examination of the excised sinus tract (Figures 3, 4) showed caseous epithelioid granulomatous inflammation with Langhans multinucleated giant cells, and the stain for AFB was positive.

Case 3

**Figure 5:**
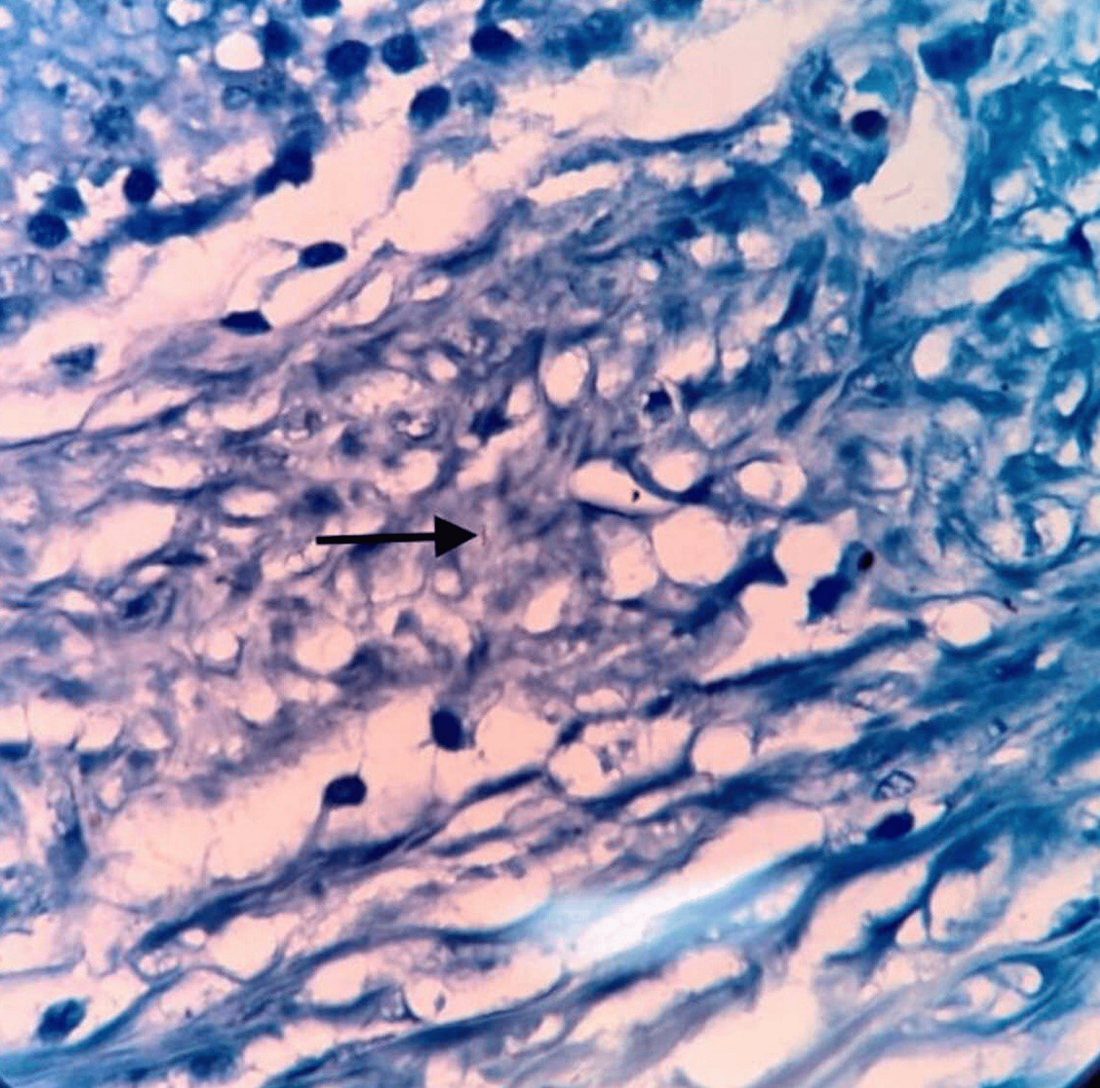
Oil immersion with 100×-modified Ziehl-Neelsen stain showing Acid Fast Bacilli (black arrow)

A 60-year-old male patient presented with a history of swelling in the left scrotum for five months with fever and night sweats. The patient had a history of pulmonary TB 35 years back and was treated and declared cured. CT KUB contrast showed a well-defined peripheral enhanced lesion measuring 2.1 × 1.8 cm in the left testis with central areas of necrosis. An orchidectomy specimen of the left testis (Figure 5) showed epithelioid granulomatous inflammation with Langhans giant cells and tubercle bacilli seen on AFB smear.

## Discussion

Urogenital TB accounts for roughly 4% of all EPTB cases each year in India. Kidneys are the most commonly affected organ. TB affecting testes is extremely rare, accounting for only 3% of male genital TB [5-8]. This may underrepresent the actual number of cases due to the challenges involved in diagnosing the condition [1]. Testicular TB is believed to result in the spread of TB bacilli through the blood and lymphatics, which is the most common route. It might also occur due to the spread of bacilli in the urinary tract into the prostate, from there into seminal vesicles and epididymis in men [9-11].

Patients with genital TB seldom exhibit systemic symptoms, such as fever, weight loss, and night sweats [12,13]. Patients have a wide variety of presentations, making the diagnosis difficult. The majority of cases present with painless or painful scrotal masses, bilateral involvement one-third of the time, and in more advanced diseases, abscess and sinus formation are noted. The presence of an abscess or sinus might indicate progression to a severe form of epididymo-orchitis. There may also be irritative lower urinary tract symptoms, such as dysuria, frequency, or urgency. The presence of constitutional symptoms of pulmonary TB or a history suggestive of active or chronic TB is important, although uncommon, in consideration of possible genital TB. In the absence of such history, consideration of possible tuberculous epididymo-orchitis requires a high index of suspicion. Hence, patients with swelling and pain in the scrotum for more than two weeks and those who do not respond to antibiotics should be assessed for genital TB. All patients with suspected male genital TB must be evaluated for urinary TB [1,9,14]. Some serious adverse outcomes of genital TB include infertility in males due to a decrease in motility and the number of sperms because of atrophy and duct obstruction [15,16]. Some might exhibit only infertility, necessitating a high level of suspicion to diagnose the condition.

Diagnosis of urogenital TB includes various modalities, such as urine microscopy, Ziehl-Neelsen technique, culture, Gene Xpert, ultrasound KUB, contrast-enhanced CT, and fine-needle aspiration cytology (FNAC). Histopathological biopsy can augment the diagnostic accuracy [17,18].

Pathological examination and culture with isolation of mycobacteria are the gold standard confirmatory tests for TB diagnosis. The specimen to be tested was acquired either through FNAC or through surgical biopsy. FNAC can provide a histological diagnosis when a clinical and radiological suspicion of malignancy is unlikely. The presence of epithelioid cell granuloma with multinucleated giant cells and caseation is diagnostic [14], even though there is no consensus on its use since others argue against the probability of increasing the risk of needle site inoculation and fistula formation.

The differential diagnosis of male genital TB includes acute bacterial infections, epididymo-orchitis, and testicular tumors. Hence, thorough clinical evaluation and investigations must be done to rule out any testicular tumors that may mimic TB [19,20]. Delay in diagnosis or misdiagnosis can lead to the dissemination of TB, infertility in young adults, and unnecessary surgical burden.

Anti-TB therapy (ATT) is the mainstay in the management of EPTB, which includes two months of intensive phase with HRZE and four months of continuous phase with HRE [20,21]. Surgery is generally unnecessary and not routinely done. However, surgical intervention may be needed if a sinus or caseating abscess persists despite ATT.

## Conclusions

Male genital TB is often underdiagnosed. This is attributed to the disease’s non-specific nature. TB should be considered a differential diagnosis in patients presenting with testicular mass and young patients with infertility. Since it is a curable disease with medical management, a high level of clinical suspicion is crucial for early diagnosis and for preventing long-term complications.
